# Cell-Autonomous Gβ Signaling Defines Neuron-Specific Steady State Serotonin Synthesis in *Caenorhabditis elegans*

**DOI:** 10.1371/journal.pgen.1005540

**Published:** 2015-09-24

**Authors:** Lu Xu, Sunju Choi, Yusu Xie, Ji Ying Sze

**Affiliations:** Department of Molecular Pharmacology, Albert Einstein College of Medicine, Bronx, New York, United States of America; Harvard University, UNITED STATES

## Abstract

Heterotrimeric G proteins regulate a vast array of cellular functions via specific intracellular effectors. Accumulating pharmacological and biochemical studies implicate Gβ subunits as signaling molecules interacting directly with a wide range of effectors to modulate downstream cellular responses, in addition to their role in regulating Gα subunit activities. However, the native biological roles of Gβ-mediated signaling pathways in vivo have been characterized only in a few cases. Here, we identified a Gβ GPB-1 signaling pathway operating in specific serotonergic neurons to the define steady state serotonin (5-HT) synthesis, through a genetic screen for 5-HT synthesis mutants in *Caenorhabditis elegans*. We found that signaling through cell autonomous GPB-1 to the OCR-2 TRPV channel defines the baseline expression of 5-HT synthesis enzyme tryptophan hydroxylase *tph-1* in ADF chemosensory neurons. This Gβ signaling pathway is not essential for establishing the serotonergic cell fates and is mechanistically separated from stress-induced *tph-1* upregulation. We identified that ADF-produced 5-HT controls specific innate rhythmic behaviors. These results revealed a Gβ-mediated signaling operating in differentiated cells to specify intrinsic functional properties, and indicate that baseline TPH expression is not a default generic serotonergic fate, but is programmed in a cell-specific manner in the mature nervous system. Cell-specific regulation of TPH expression could be a general principle for tailored steady state 5-HT synthesis in functionally distinct neurons and their regulation of innate behavior.

## Author Summary

Levels of neurotransmitter serotonin synthesis shape disparate behaviors in evolutionary diverse organisms, but the mechanisms defining steady state serotonin synthesis in functionally distinct neuronal types remain unknown. A genetic screen for neuron-specific serotonin synthesis mutants in *Caenorhabditis elegans* revealed a unique Gβ GPB-1 signaling pathway operating in specific serotonergic neurons to define the baseline expression of serotonin synthesis rate-limiting enzyme tryptophan hydroxylase *tph-1*. Unlike in canonical heterotrimeric G protein signaling pathways where Gα subunits drive downstream effectors, we found that signaling through Gβ GPB-1 to the OCR-2 TRPV channel defines the baseline *tph-1* expression. This Gβ signaling is not required for the establishment or maintenance of the serotonergic cell fates, but dedicated to set steady state 5-HT synthesis in mature neurons. Behavioral analyses showed that 5-HT synthesized in different neurons modulates distinct innate rhythmic behaviors. Our work identified a Gβ-mediated signaling pathway operating in differentiated neuronal cells to specify intrinsic functional diversities, and illuminate a mechanistic principle for genetic programming of neuron-specific steady state 5-HT synthesis in dedicated behavioral circuits.

## Introduction

Serotonin (5-HT) is a neuromodulator implicated in stress-triggered behaviors such as aggression, anxiety, as well as in diverse innate behaviors and physiological processes ranging from food intake to rhythmic motor acts and circadian cycles [1]. Increasing evidence suggests that too much or too little of 5-HT signals from particular neurons contribute to aspects of behavioral and physiological alterations [1,2]. Heterogeneity of 5-HT-producing neurons has been characterized in human and rodent central nervous system (CNS) based on anatomical distributions, axonal trajectories and electrophysiological properties [3]. While the signaling pathways specifying serotonergic cell fates have been studied extensively, little is known about the genetic program defining 5-HT production in mature nervous systems.

Studies in rodents suggest that transcriptional regulatory networks modulate CNS 5-HT synthesis throughout an animal’s life [2,4,5]. In the current paradigm, levels of 5-HT synthesis under favorite environment are considered the steady state or baseline 5-HT signals, and internal and external stressors may further enhance 5-HT synthesis for facilitating behavioral and physiological adaptation [4]. It is generally assumed that the baseline 5-HT synthesis is a feature of default serotonergic cell fates [6]. This view, however, does not address how basal levels of *Tph2*, encoding the CNS 5-HT synthesis rate-limiting enzyme tryptophan hydroxylase, display stereotyped spatiotemporal changes after the 5-HT cell fate established [7]. Further, early life experience can influence baseline *Tph2* expression in the adulthood CNS [4]. Alternative theories propose that additional regulatory programs define 5-HT synthesis [5]. Indeed, the transcription factor PET-1 maintains *Tph2* expression in 70% of CNS 5-HT neurons in adult mice to influence anxiety behavior [8]. Thus, steady state 5-HT synthesis could set the tone of innate neural circuitry and influence the sensitivity to stress-induced neural plasticity. However, the genetic programs that define steady state 5-HT synthesis in functionally distinct neurons and its mechanistic relation to stress-induced changes in 5-HT synthesis in an animal remain to be discovered.

We have focused on genetic dissection of serotonergic phenotypes in *C*. *elegans*. The small *C*. *elegans* nervous system has a classical 5-HT system with the characteristic diversities. Of 302 neurons in a hermaphrodite worm, three pairs, ADF chemosensory neurons, NSM pharyngeal secretory neurons and HSN motor neurons, produce 5-HT [9]. Each pair has unique morphological and functional features but expresses a common set of serotonergic phenotype genes: the sole tryptophan hydroxylase gene *tph-1*, vesicular monoamine transporter (VMAT) *cat-1* for 5-HT release and 5-HT uptake transporter SERT *mod-5*. Using a green fluorescent protein reporter for *tph-1* (*tph-1*::*gfp*), our laboratory and others have identified a variety of internal and external stresses via distinct signaling pathways upregulating *tph-1* expression in specific neurons to influence behavior, emphasizing *tph-1* transcription as a key to dynamic changes in 5-HT signaling [10–13].

The β subunits of the heterotrimeric guanine nucleotide-binding proteins (G proteins) have been implicated in multiple aspects of G protein signaling. Gβ proteins are members of a large family of proteins containing multiple copies of tryptophan-aspartic acid repeat (WD40) motifs that may interact with diverse partners [14]. In canonical G protein signaling pathways, Gβ serves as a negative regulator of Gα interaction with downstream effectors that drive cellular responses [15]. Evidence has been accumulating that Gβ proteins also directly signal to their own effectors, in both Gα-coupled receptor-dependent and receptor-independent mechanisms [16,17]. Co-crystal structure determination and NMR analysis revealed several effector-binding hotspots on the Gβ WD40 repeats domain, and biochemical studies demonstrated that purified Gβ may induce diverse cellular responses by directly interacting with ion channels, enzymes, and arrays of signaling molecules [16,17]. Indeed, pharmacological analyses and cell-based studies have implicated Gβ-mediated signaling in cell cycles, cellular differentiation and stress responses [16–18]. In general, however, the physiological roles of Gβ signaling in native cellular contexts remain to be elucidated.

In this paper, we report a cell-autonomous Gβ signaling defining baseline *tph-1* expression. A genetic screen for 5-HT synthesis mutants identified a Gβ GPB-1 to TRPV channel OCR-2 signaling pathway that is necessary and sufficient to define baseline *tph-1* expression, thus 5-HT synthesis, in ADF neurons. This GPB-1-mediated signaling is not required for establishment or maintenance of the cell fates, and is mechanistically separated from stress-induced *tph-1* upregulation. Our data establish a role for Gβ-mediated signaling operating in mature neurons to specify the intrinsic functional properties. Gβ-mediated signaling could represent a genetic determinant underlying neuron-specific steady state 5-HT synthesis in functionally distinct neurons that regulate specific aspects of innate behavior.

## Results

### GPB-1 specifically regulates *tph-1* expression in ADF neurons

**Fig 1:**
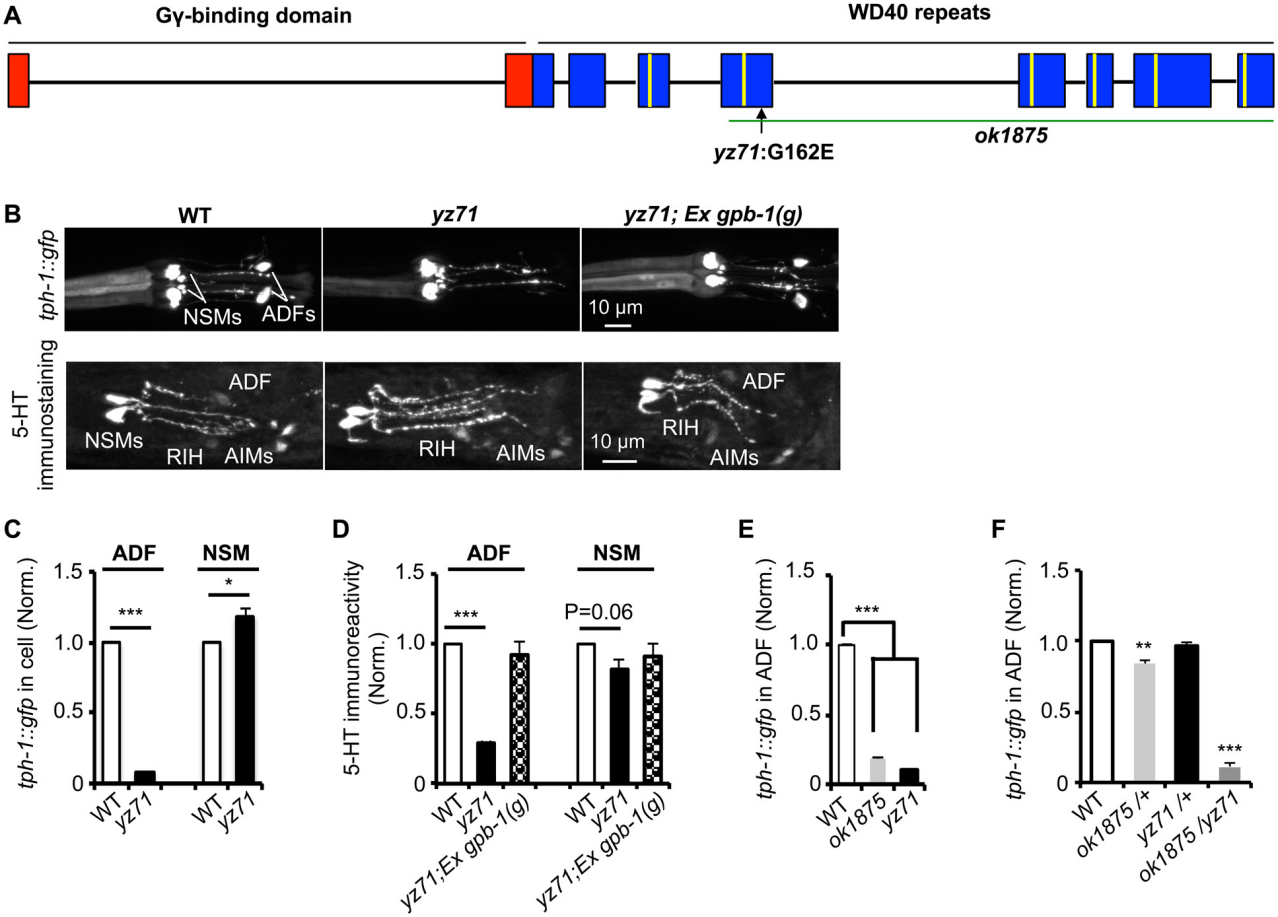
*gpb-1* deficient mutants specifically diminish 5-HT synthesis in ADF neurons. **(A)** *gpb-1* mutant alleles. Lines denote introns and rectangles denote exons of *gpb-1* gene. The predicted region binding to Gγ (red) and seven WD40 repeats of the propeller domain (blue) that interacts with Gα are in reference of previous work [28,53]. A green line indicates the deleted region in the *ok1875* allele, and an arrow points to the location of the *yz71* mutation. **(B)** Photomicrographs showing *tph-1*::*gfp* and 5-HT immunostaining were selectively diminished in ADF neurons in *yz71* mutants and rescued by *gpb-1(g)* transgene. **(C—D)** Quantification of *tph-1*::*gfp* fluorescence (**C**) and 5-HT immunoreactivity (**D**) in ADF and NSM neurons in L4 stage animals. **(E)** Quantification of ADF *tph-1*::*gfp* in WT, *gpb-1(ok1875*)-null and *yz71* at L1 stage. **(F)** Comparing ADF *tph-1*::*gfp* in WT with that in *ok1875/yz71* hemizygotes, and *ok1875/+* and *yz71/+* heterozygotes. Data represent the average of 2 (5-HT staining) or 3 (GFP) repeats. N = 18–32/genotype/trial. The value of fluorescence of mutants and transgenic animals is normalized to that of WT animals, mean ± SEM, *p < 0.05, **p < 0.01, ***, p < 0.001, t-tests.

We isolated the *yz71* mutant through an unbiased forward genetic screen for mutations that specifically diminished *tph-1*::*gfp* expression in ADF serotonergic chemosensory neurons [12]. Under optimal growth conditions, *yz71* mutants showed an ~90% reduction in ADF *tph-1*::*gfp* throughout the life, as compared to wild-type (WT) animals (Fig 1B and 1C). In contrast, *tph-1*::*gfp* in other neurons was not reduced in *yz71* mutants (Fig 1B and 1C). 5-HT immunostaining of *yz71* mutants showed dramatically diminished 5-HT levels in ADF (Fig 1B and 1D). We therefore concluded that *yz71* impairs a gene function specifically required for *tph-1* expression, thus 5-HT synthesis, in the ADF neurons.

Single nucleotide polymorphism (SNP)-based mapping narrowed the *yz71* mutation to a 700 kb contig of LG II, where we identified a missense mutation causing a G162E substitution in the third WD40 repeat of the Gβ protein GPB-1 (Fig 1A). We confirmed that GPB-1(G162E) is responsible for diminished 5-HT in *yz71* mutants, by determining that transgenic expressing WT genomic *gpb-1* sequence restored ADF *tph-1*::*gfp* and 5-HT immunostaining (Fig 1B and 1D).

To probe G162E effects on GPB-1 functionality, we analyzed a *gpb-1* deletion allele, *ok1875*. Homozygous *gpb-1* null alleles are lethal [19], but those carrying maternal GPB-1 from heterozygous hermaphrodites can survive to larval stage 1 (L1) and displayed reduced ADF *tph-1*::*gfp* (Fig 1E). Importantly, a majority of *yz71/ok1875* hemizygotes also died at L1, and the few that survived displayed reduced ADF *tph-1*::*gfp* (Fig 1F). In contrast, ADF *tph-1*::*gfp* levels in both *yz71/+* and *ok1875/+* heterozygotes were close to WT (Fig 1F). Taken together, these data identified that *gpb-1* selectively regulates ADF *tph-1* expression, and G162E is a recessive, reduction-of-function *gpb-1* allele. Unlike *gpb-1* null alleles, *yz71* homozygotes are fertile, providing a genetic system for characterizing native GPB-1 function in an animal.

### GPB-1(G162E) does not impair serotonergic cell fates

As GPB-1 functions early in embryogenesis [19,20], an immediate question was whether ADF neurons are present in *yz71* mutants. In the mammalian CNS, postmitotic precursors of 5-HT neurons are born, migrate and acquire other neuronal features hours to days before committing to express 5-HT phenotype genes [2]. In *C*. *elegans*, ADFs are generated and migrate to the amphid sensilla during embryogenesis and switch on *tph-1* expression after hatching [9,21]. We therefore investigated if *gpb-1(yz71)* would disrupt ADF formation, survival, or its serotonergic fate.

**Fig 2:**
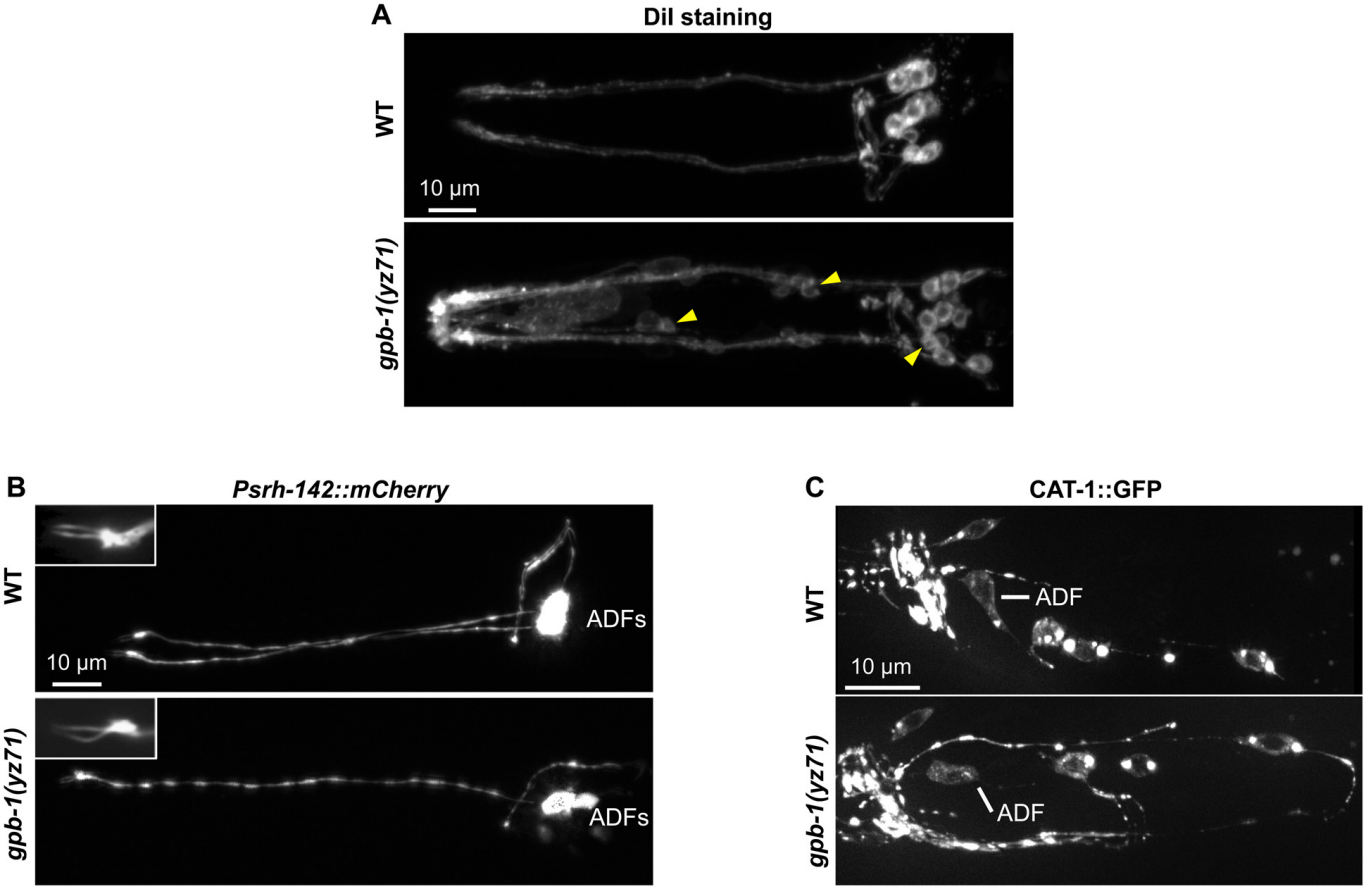
*gpb-1(yz71)* does not abrogate ADF cell fates. **(A)** Visualizing amphid neuronal architecture in living animals using DiI filling. In *yz71* mutants, DiI filled into the typical 6 pairs of amphid neurons, although the dye could sometimes be detected in several additional neurons (arrowheads). **(B)** ADF-specific marker *Psrh-142*::*mCherry* was expressed and showed characteristic ADF cilia structure (insets) in *yz71* mutants. **(C)** GFP-tagged VMAT CAT-1 protein was expressed in ADF of *yz71* mutants. Animals at all developmental stages were examined, and images of L4 animals are shown.

We first determined whether amphid sensory neurons were generated in *gpb-1(yz71)* mutants, using dye DiI filling. The pair of amphids in the head is the major *C*. *elegans* sensory organ, each comprising one ADF and eleven other classes of non-serotonergic sensory neurons with characteristic ciliated sensory endings sensing particular environmental cues [22]. We observed DiI filled into the typical six pairs of the amphid neurons in *yz71* mutants as in WT animals (Fig 2A). Judged by DiI fluorescence, the overall neuronal organization and their axonal and dendritic morphology were preserved in *yz71* mutants (Fig 2A). However, while DiI rarely filled into ADF neurons in WT animals (8%, N = 26), 31% of ADF were filled with DiI in *yz71* mutants (N = 65). DiI also filled into a few non-amphid neurons in some *yz71* mutants (Fig 2A).

We next determined whether *yz71* altered gross ADF cell fates, using a mCherry reporter for the ADF-specific marker gene *srh-142*. Fig 2B shows that *Psrh-142*::*mCherry* was expressed in *yz71* mutants, and their ADFs displayed the characteristic axon, dendritic and cilia architectures.

To assess the general serotonergic cell fate, we used a functional GFP reporter for VMAT CAT-1 protein [23]. We did not detect an appreciable change in GFP-tagged CAT-1 expression or subcellular localization in *yz71* mutants (Fig 2C). These data showed that ADF neurons were generated and maintained serotonergic fates in *yz71* mutants, leading to our hypothesis that GPB-1 selectively mediates transcriptional control of 5-HT synthesis.

### Deficits in baseline and stress-induced *tph-1* expression are mechanistically separable

ADF *tph-1* expression is highly sensitive to external and internal environment. Gβ of heterotrimeric G proteins could function in multiple signaling pathways in concert with different Gα subunits [16]. Therefore, GPB-1 function could define a mechanism setting baseline *tph-1* expression, as well as be involved in signaling pathway(s) that mediate stress-induced *tph-1* upregulation. To discern and define the role of GPB-1 in steady state and stress-induced 5-HT synthesis, we measured *tph-1*::*gfp* in *yz71* mutants under optimal conditions and two well-established aversive conditions, dauer formation and pathogenic bacterial infection.

**Fig 3:**
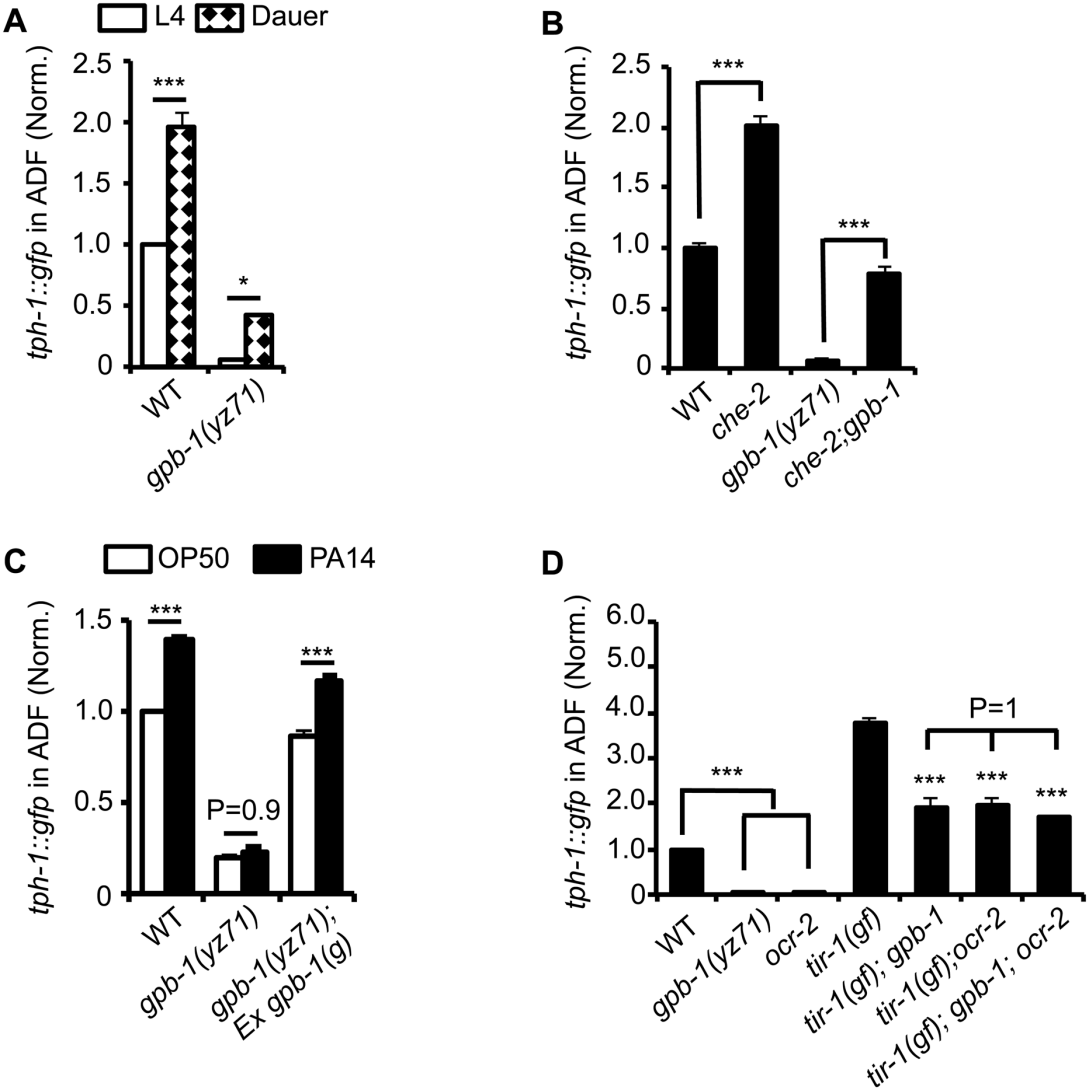
*tph-1*::*gfp* expression in *gpb-1(yz71)* mutants under optimal and aversive conditions. **(A)** Dauer formation induced by aversive growth conditions caused ADF *tph-1*::*gfp* upregulation in WT and *yz71*mutants, as compared to their respective L4 stage siblings. **(B)** Mutation in cilia structural gene *che-2* was capable of triggering ADF *tph-1*::*gfp* upregulation in *yz71* mutants. **(C)** Pathogen PA14 failed to induce ADF *tph-1*::*gfp* upregulation in *yz71* mutants, as comparing *tph-1*::*gfp* between 1^st^ day adults fed PA14 and non-pathogenic bacterial control OP50 for 6 hr. *gpb-1(g)* transgene restored the PA14 response in *yz71* mutants. **(D)** Comparing ADF *tph-1*::*gfp* between *gpb-1(yz71)* and *ocr-2* TRPV channel mutants. In both mutants, ADF *tph-1*::*gfp* was diminished under optimal growth conditions, but enhanced in *tir-1(yz68gf)* background as compared to the *yz71* and *ocr-2* single mutants. *yz71* and *ocr-2* did not produce an additive effect. For each assay, the value of ADF GFP fluorescence in WT animals under a stress paradigm and that of mutants is normalized to the value of WT animals under optimal conditions. Data represent the average of ≥ 3 trials ± SEM, * p < 0.05, *** p < 0.001, t-test for two group comparisons, and ANOVA for multi-group/condition comparisons.

A stress-resistant state called dauer can be induced by a defined set of aversive growth conditions and is the most commonly used paradigm in *C*. *elegans* stress response studies [24]. Dauers enhance a battery of stress responsive genes to produce a series of physiological, morphological and behavioral remodeling [24]. Previously, we identified that alteration of ADF sensory cilia architecture caused either by dauer formation or genetic mutations in intraflagellar transport (IFT) triggers ADF *tph-1* upregulation [10]. When *yz71* mutants were induced to form dauers, ADF *tph-1*::*gfp* was significantly enhanced compared to their siblings under optimal growth conditions (Fig 3A). Likewise, we observed elevated ADF *tph-1*::*gfp* in *yz71* mutants carrying defective IFT component *che-2* (Fig 3B). However, ADF *tph-1*::*gfp* levels in *yz71* dauers and *yz71*;*che-2* double mutants were lower than WT dauers and *che-2* single mutants, respectively (Fig 3A and 3B). These observations support the idea that dauer formation and GPB-1 regulate two mechanistically separable layers of *tph-1* expression. *yz71* mutants do not impair *tph-1* upregulation induced by dauer formation or cilia structural alterations but have a lower baseline of *tph-1* expression.

Pathogenic bacterial infection is another established aversive paradigm inducing ADF *tph-1* upregulation. Feeding worms the human opportunistic pathogen *Pseudomonas aeruginosa* strain PA14, instead of nutritious *E*. *coli* OP50, specifically induces ADF *tph-1* upregulation to facilitate behavioral avoidance of the pathogenic food [11]. PA14-induced ADF *tph-1*::*gfp* upregulation requires G_q_α protein EGL-30 [25]. Consistent with Gβ as a regulator of Gα signaling, *yz71* mutants failed to upregulate ADF *tph-1*::*gfp* during PA14 infection, and the *gpb-1(g)* transgene rescued the PA14 sensitivity (Fig 3C). However, whereas both *egl-30(lf)* and *egl-30(gf)* abrogated PA14-induced *tph-1*::*gfp* upregulation, neither the *egl-30* mutation dramatically diminished ADF *tph-1*::*gfp* [10,25], suggesting that the baseline and pathogen-induced *tph-1* expression are mediated by mechanistically separated GPB-1 functions.

Previous work established that PA14 triggers ADF *tph-1* upregulation via a cell-autonomous Toll-interlukin-1 receptor domain adaptor protein TIR-1 activated MAPK signaling pathway [12,26]. As another method to distinguish GPB-1 function between the baseline and pathogen-induced *tph-1* expression, we generated double mutants of *yz71* and *tir-1(yz68gf)*. *tir-1(gf)* mutants constitutively enhance ADF *tph-1*::*gfp* by activating the MAPK signaling [12]. If GPB-1 functions purely in the pathogen-responsive pathway, *tir-1(gf);yz71* double mutants would display ADF *tph-1*::*gfp* levels similar to one of the single mutants. In contrary, *tir-1(gf);yz71* double mutants displayed an intermediate ADF *tph-1*::*gfp* level between the two single mutants (Fig 3D). This result suggests that GPB-1 functions upstream of TIR-1 in the pathogen responsive pathway, while a TIR-1-independent GPB-1 function defines baseline *tph-1* expression.

We further tested this hypothesis by generating double mutants of *tir-1(gf)* and *ocr-2*. Disruption of cell-autonomous OCR-2 TRPV channel specifically diminishes ADF baseline *tph-1* expression [12,27]. If *yz71* disrupts the OCR-2 pathway, ADF *tph-1*::*gfp* levels in *tir-1(gf);ocr-2* and *tir-1(gf);yz71* should be similar. This was what we observed (Fig 3D). Further, *tir-1(gf);gpb-1(yz71);ocr-2* triple mutants did not display a lower ADF *tph-1*::*gfp* level compared to the double mutants (P = 1, ANOVA) (Fig 3D). Combined, these data suggest that GPB-1 function is essential for EGL-30 G_q_α signaling during pathogen infection, and an EGL-30- and TIR-1-independent GPB-1 function in the OCR-2 signaling pathway directs baseline ADF *tph-1* expression.

### Cell-autonomous GPB-1 directs baseline *tph-1* expression

GPB-1 is expressed broadly in neurons and non-neuronal tissues [19]. To identify and discern the GPB-1 action sites for basal and pathogen-induced *tph-1* expression, we generated transgenic lines expressing *gpb-1* cDNA in defined cells and tested their ability to rescue ADF *tph-1*::*gfp* expression in *yz71* mutants under optimal conditions and during PA14 infection.

**Fig 4:**
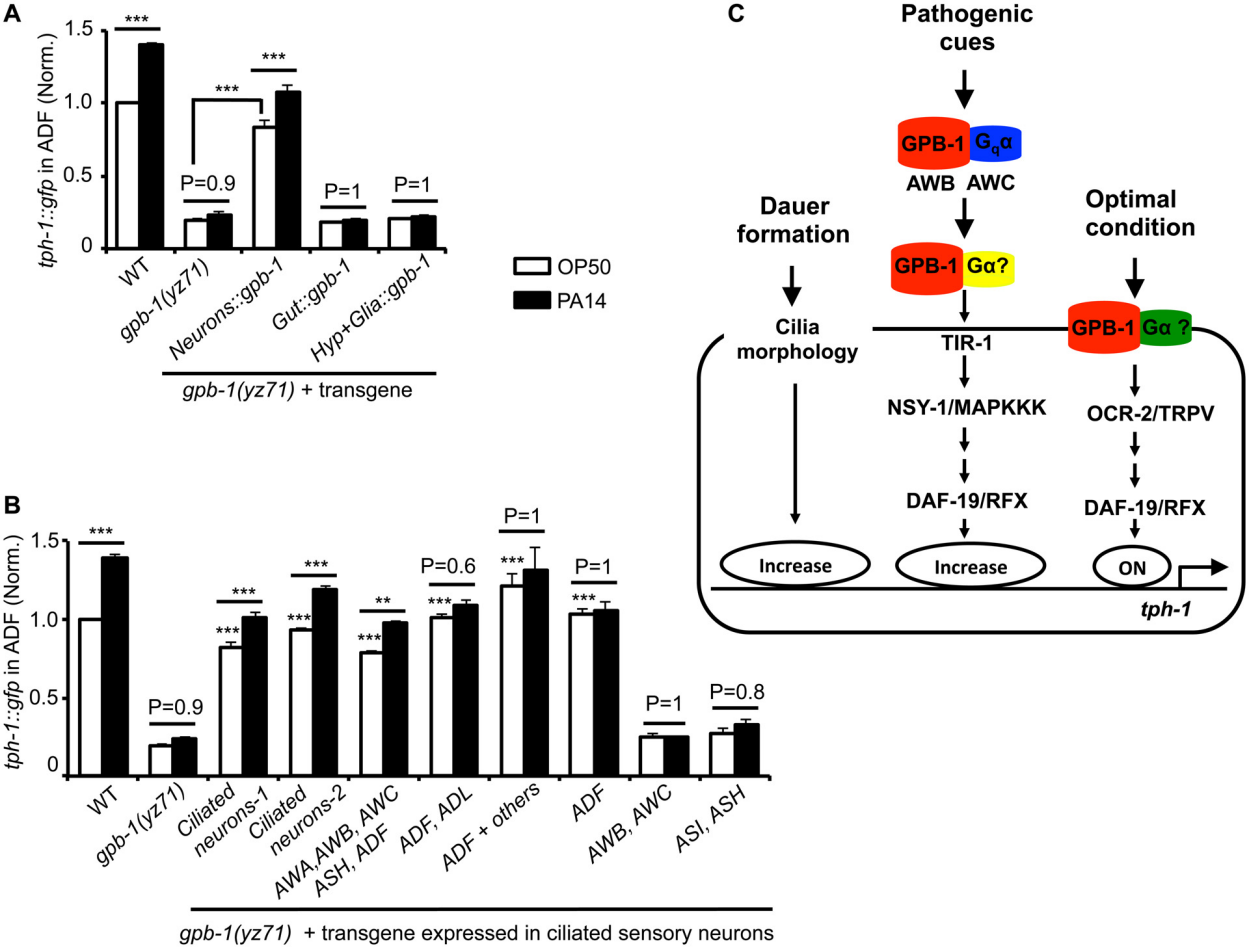
Baseline and pathogen-induced ADF *tph-1*::*gfp* expression involve separated GPB-1 functions. **(A)** Transgenic expressing *gpb-1* cDNA in all neurons rescued both baseline and pathogen PA14-induced ADF *tph-1*::*gfp* expression in *yz71* mutants. **(B**) Expressing *gpb-1* cDNA specifically in ADF restored baseline *tph-1*::*gfp* but not the response to PA14. ADF is the only neuronal type expressed in common by the three transgenes that selectively rescued the baseline ADF *tph-1*::*gfp* (the ADF and ADL expression was driven by a *lin-11* promoter, and the ADF + others expression was driven by the *cat-1* promoter). The transgenes expressed in additional amphid neurons including ADF rescued both the baseline and PA14-induced *tph-1*::*gfp* expression, but the transgenes that are not expressed in ADF failed to rescue under either condition. For each assay, the value of ADF GFP fluorescence in WT animals fed PA14 and that of mutants and transgenic animals is normalized to the value of WT animals under optimal conditions. Data represent the average of ≥ 2 trials ± SEM. The differences between *yz71* mutants and the transgenic animals under optimal growth conditions are marked on the top of white bars, and the differences between the same strains fed OP50 and PA14 are indicated, ** p < 0.01, *** p < 0.001, ANOVA followed by Tukey tests. **(C)** Model for GPB-1 regulation of the baseline and pathogen-induced *tph-1* expression in ADF. Cell-autonomous GPB-1 is necessary and sufficient for the baseline *tph-1* expression. GPB-1 regulates PA14-induced *tph-1* upregulation by controlling G_q_α EGL-30, which acts in AWB and AWC neurons in the pathogen-induced signaling pathway [25], and other Gα signaling in additional amphid neurons.

We first expressed GPB-1 in all neurons, or in hypodermal and glial cells surrounding amphid sensory cilia, or in the gut. Fig 4A shows that expressing GPB-1 in neurons, but not in the other tissues, rescued both the basal and PA14-induced *tph-1*::*gfp* expression.

As G_q_α EGL-30 functions in AWB and AWC amphid neurons to mediate PA14-induced *tph-1*::*gfp* upregulation in ADF [25], we tested whether GPB-1 acts in AWB and AWC to direct both the basal and PA14-induced *tph-1* expression in ADF. Surprisingly, expressing GPB-1 in AWB and AWC failed to rescue *tph-1*::*gfp* under either condition (Fig 4B). In contrast, expressing GPB-1 in all the ciliated neurons or selectively in AWA, AWB, AWC, ASH and ADF restored ADF *tph-1*::*gfp* under both the conditions (Fig 4B). To probe a cell-autonomous role for GPB-1 in ADF, we generated two transgenes that share in common only in expressing GPB-1 in ADF and a third transgene expressing GPB-1 specifically in ADF. All the three transgenes robustly rescued the basal ADF *tph-1*::*gfp* expression, but none responded to PA14 (Fig 4B). We concluded that pathogen-induced *tph-1* upregulation requires GPB-1 in AWB, AWC and additional amphid sensory neurons. In contrast, cell autonomous GPB-1 function is necessary and sufficient to define the baseline *tph-1* expression in the ADF neurons (Fig 4C).

### G162E alters a conserved residue at the interface between Gβ and switch II helix of Gα

**Fig 5:**
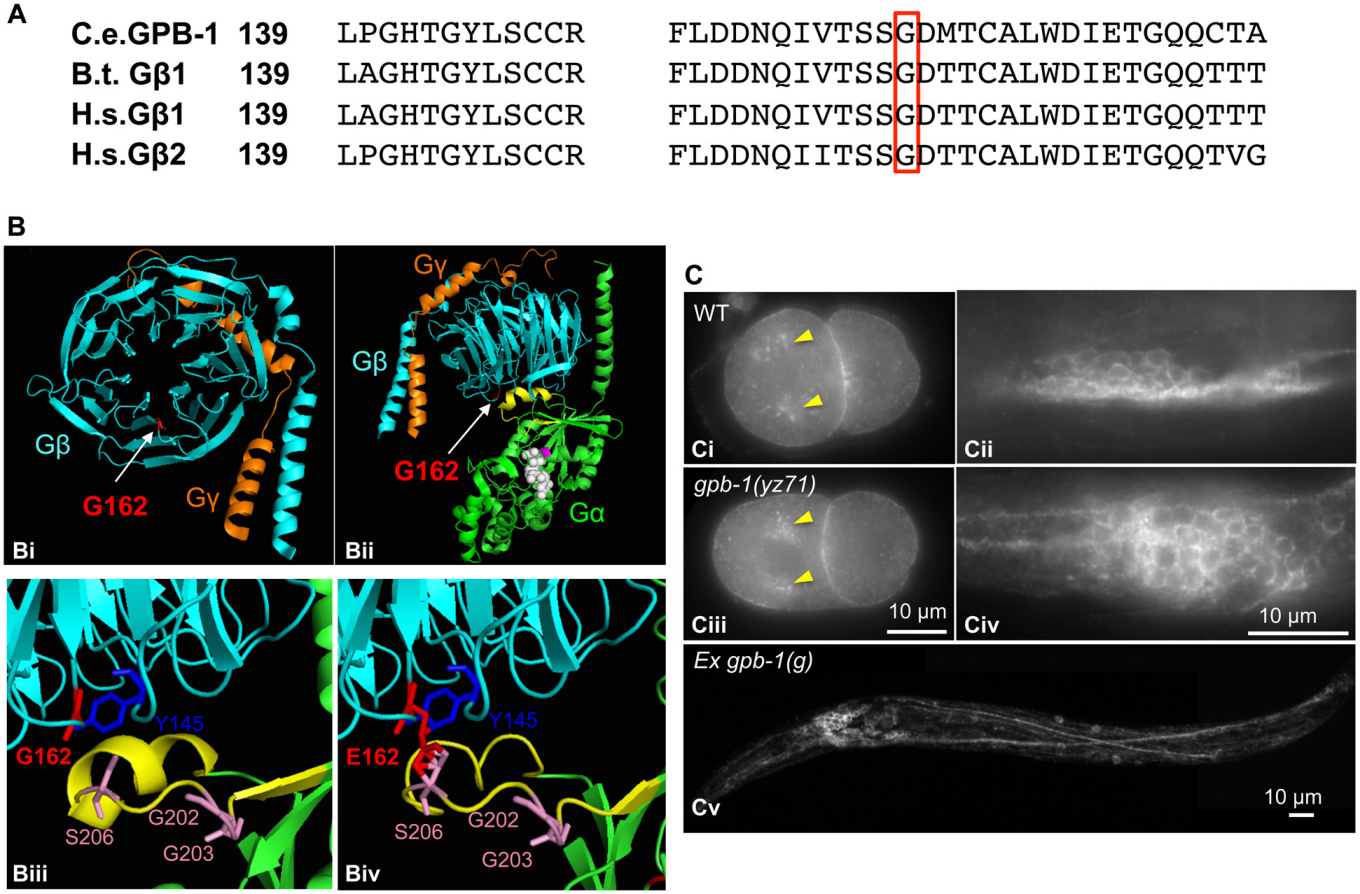
G162E occurs at the Gβ surface that binds to Gα switch II helix and Gβ effectors. **(A)** Comparing amino acid sequence surrounding G162 (red boxed) between *C*. *elegans* GPB-1, bovine (B.t.) Gβ1, and human (H.s.) Gβ1 and Gβ2, all of which contain 340 amino acid residues and the numbers refer to residue positions in the corresponding proteins. **(B) Modeling G162E substitution over bovine β1. (Bi)** Cartoon presentation of the crystallographic structure of rat G_i_α_1_ and bovine β1γ2 heterotrimer rendered with PyMOL software. β subunit is colored cyan, and γ subunit colored orange. G162 (the side chain colored red) is located on the surface of the narrow side of the propeller architecture of Gβ. **(Bii)** Another view of the heterotrimer showing the interactions between Gβ and GDP-bound Gα (green). G162 is located on the surface that binds to the Gα switch II helix (yellow). GDP is in white, and the nearby Gα-S47 that is critical for the GTP binding is colored magenta. **(Biii)** An enlarged view showing the interface between Gβ and Gα switch II helix. Gβ-G162 sits nearby Gβ-Y145, a critical residue in an effector binding hotspot [17]. Gβ-G162 is in close proximity to Gα-S206 in the switch II helix. The conformation of Gα-G202 and G203 is critical for GTP binding [32]. **(Biv)** An enlarged view showing the Gβ and Gα switch II interface, with G162 changed to E in the Gβ sequence. **(C)** GPB-1 immunostaining. **Ci** and **Ciii**. In both WT and *yz71* two-cell embryos, GPB-1 was enriched in the asters (arrowheads) and in the region between cells. **Cii** and **Civ**. In WT and *yz71* animals, GPB-1 can be detected in the cell membrane in neurons. **Cv**. *gpb-1(g)* overexpression transgenic animals showing GPB-1 localized to axons and dendrites.

How might GPB-1(G162E) disrupt G protein functionalities? GPB-1 shares 86% amino acid identity with human and bovine Gβ1 (Fig 5A). To gain insights into GPB-1 functional mechanisms regulating *tph-1* expression, we modeled GPB-1(G162E) over the crystallographic structure of rat G_i_α1 and bovine β1γ2 heterotrimer [28]. The seven WD40 repeats of Gβ fold into a circular β-bladed propeller which makes two major contacts with Gα: the side of the propeller interacts with the Gα N-terminal helix, and the narrow propeller region forms an interface with the Gα switch II helix [28] (Fig 5Bi and 5Bii). Formation of Gβ/Gα switch II interface favors the inactive GDP-binding state, whereas GTP binding of Gα triggers the switch II helix rotating 120^°^ thereby permitting both Gβ and Gα proteins to interact with their effectors [28,29]. G162E occurs right at the Gβ/Gα switch II interface (Fig 5Biii and 5Biv).

Because *gpb-1(yz71)* is a reduction-of-function allele, G162E is likely to disrupt Gβ gene function. Whole mount GPB-1 immunostaining indicated that G162E does not grossly impair GPB-1 expression or subcellular localization. For example, both *yz71* and WT 2-cell-stage embryos showed GPB-1 enriched in the aster and cell membranes between cells (Fig 5Ci and 5Ciii). In *yz71* animals, as in WT and GPB-1-overexpressing animals, GPB-1 was detected in neuronal and non-neuronal tissues, and localized to the cell membrane as well as in axons and dendrites in neurons (Fig 5Cii, 5Civ and 5Cv), as seen in published GPB-1 expression patterns using different GPB-1 antibodies [19]. Because heterotrimeric complex formation is required for Gβ membrane localization [28], our data suggest that GPB-1(G162E) is likely capable of forming G protein complexes but disrupts the signaling.

### Diminished baseline *tph-1* in *gpb-1* mutants is not caused by reduced Gα signaling

**Fig 6:**
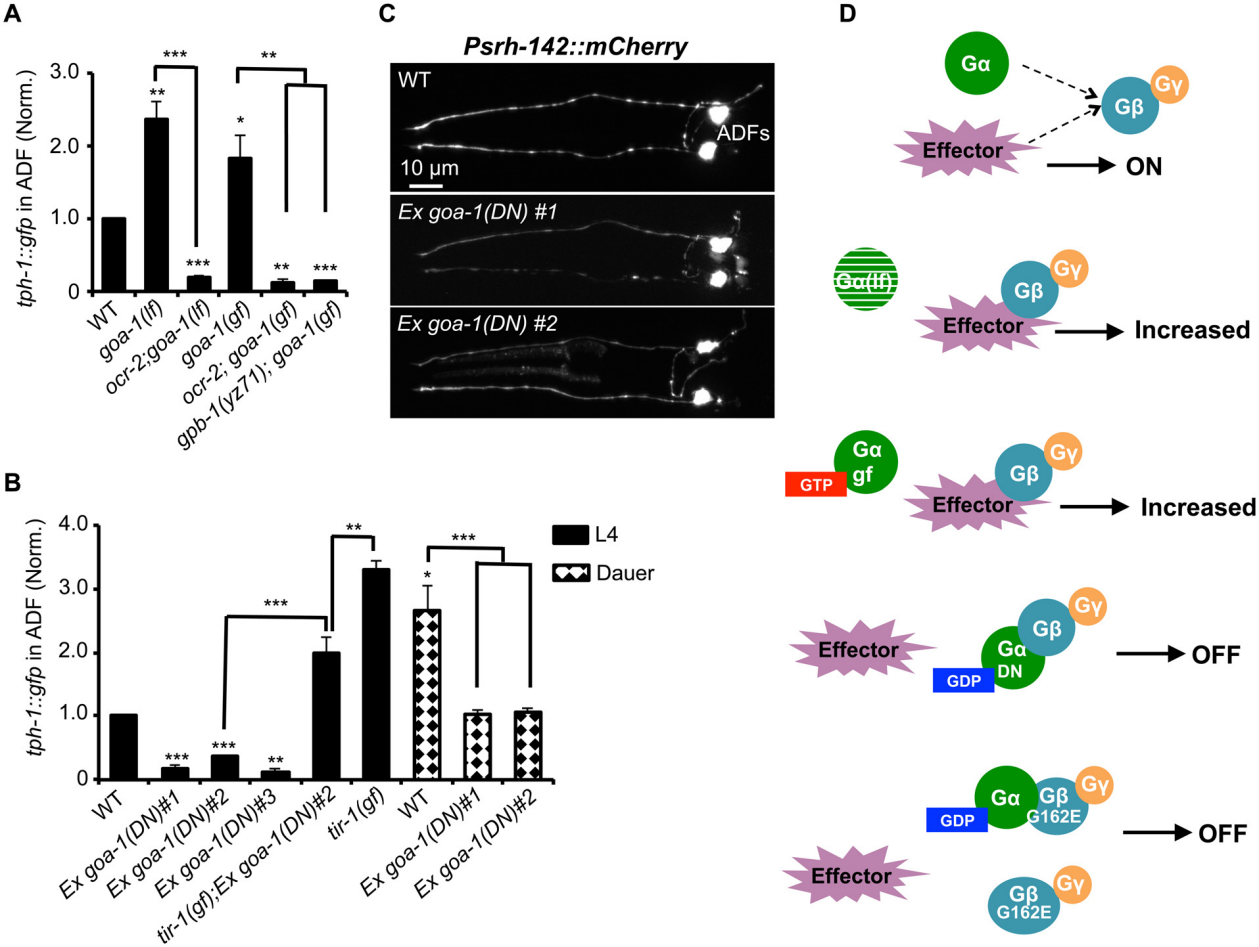
GPB-1 regulates baseline ADF *tph-1*::*gfp* expression in a GOA-1-dependent manner. **(A)** Both GOA-1 deletion (*goa-1lf*) and GTP-bound hyperactive GOA-1(Q205L) (*goa-1gf*) mutations elevated ADF *tph-1*::*gfp* compared to WT. *gpb-1(yz71)* and *ocr-2* TRPV mutations suppressed *tph-1*::*gfp* in *goa-1(lf)* and *goa-1(gf)* backgrounds. **(B)** Dominant negative GDP-bound GOA-1(S47C) transgene (*goa-1DN*) diminished ADF *tph-1* expression in three independent transgenic lines. *goa-1(DN)* transgenic animals remained capable of enhancing ADF *tph-1*::*gfp* when induced to form dauers or in *tir-1(gf)* background. For each assay, the value of ADF GFP fluorescence in dauers and that of mutants and transgenic animals is normalized to the value of WT animals under optimal conditions. Data represent the average of ≥ 2 trials ± SEM. The differences between WT and mutants are marked on the top of bar, and the differences between comparison groups are indicated, *p < 0.05, ** p < 0.01, *** p < 0.001, t-test for two group comparisons, and ANOVA for multi-group/condition comparisons. **(C)** ADF-specific marker *Psrh-142*::*mCherry* was expressed and showed characteristic ADF axon and dendritic morphology in *goa-1(DN)* transgenic animals. Animals at all developmental stages were analyzed, and images of L4 animals are shown. **(D)** A schematic of GPB-1 and GOA-1 interaction on *tph-1* expression. Effector molecules compete with G_o_α for Gβ binding to drive *tph-1* expression. In *goa-1(lf)* an *goa-1(gf)* mutants, the effector constitutively binds to Gβ. GDP-bound conformation of the *GOA-1(DN)* protein blocks the effector binding. GPB-1(G162E) obstructs the interactions with both GOA-1 and the effector, leading to constitutively diminished *tph-1* regardless of the presence of GDP- or GTP-bound G_o_α.

Gβ could serve as a regulator of Gα signaling that drives *tph-1* expression. Alternatively, Gβ itself could function as the signaling molecule to define *tph-1* expression in ADF neurons. We first considered the possibility that altered Gα-mediated signaling underscores diminished *tph-1* expression in *gpb-1* mutants. ADF expresses seven Gα genes. Loss-of-function mutants of *egl-30*, *gpa-3*, *gpa-10*, *gpa-13* and *odr-3* did not dramatically diminish ADF *tph-1*::*gfp* [10,25,27]. *gsa-1(lf)* is lethal, but RNAi of *gsa-1* also did not diminish ADF *tph-1*::*gfp* (134±14%, N = 50) compared to empty vector control (N = 28). Further, two deletion alleles of G_o_α *goa-1*, *n363* and *sa734*, both markedly elevated ADF *tph-1*::*gfp* [10,25] (Fig 6A). Repeated attempts failed to generate a *goa-1(n363);gpb-1(yz71)* double mutant. As both GOA-1 and GPB-1 function in early embryogenesis [19,20,30], eliminating GOA-1 in *gpb-1(yz71)* background could be lethal. We therefore generated a double mutant of *ocr-2* and *goa-1(n363)*, and found that *ocr-2;goa-1(n363)* double mutants displayed diminished ADF *tph-1*::*gfp* as in *ocr-2* single mutants (Fig 6A), suggesting that the absence of GOA-1 protein elevated *tph-1* expression by activating the OCR-2 TRPV pathway.

We then tested a scenario in which GPB-1(G162E) inhibits *tph-1* expression by inducing constitutive GOA-1 activation. We obtained a transgenic strain overexpressing gain-of-function GOA-1(Q205L) protein [31]. GOA-1(Q205L) is thought to constitutively activate GOA-1 signaling by locking the protein in the GTP-bound conformation [31]. Indeed, GOA-1(Q205L) retarded locomotion and egg laying, opposite to that seen with *goa-1(lf)* mutants [31]. However, contradicting to our hypothesis, GOA-1(Q205L) conferred an OCR-2-dependent increase in ADF *tph-1*::*gfp* as the *goa-1* deletion alleles (Fig 6A). Further, GOA-1(Q205L) failed to induce *tph-1*::*gfp* in *yz71* mutant background (Fig 6A), indicating that GOA-1(Q205L)-induced *tph-1* expression requires GPB-1 at work.

These results prompted us to hypothesize that both lacking GOA-1 and GTP-bound GOA-1 constitutively activate GPB-1-mediated signaling that sets baseline *tph-1* expression. To test this possibility, we constructed a dominant negative (DN) mutation in GOA-1 analogous to the mammalian G_o_α(S47C) mutation, which is locked in the GDP-bound conformation, and therefore constitutively tethers βγ and simultaneously shuts off both G_o_α and βγ mediated signaling [32]. We were unable to generate transgenic line expressing GOA-1(S47C) under the *goa-1* promoter, perhaps also because *goa-1* plays a critical role in early embryogenesis [19,20]. Therefore, we expressed GOA-1(S47C) under the VMAT *cat-1* promoter. Three independent transgenic lines all dramatically reduced ADF *tph-1*::*gfp* (Fig 6B). Like *gpb-1(yz71)* mutants (Fig 2B), the GOA-1(S47C) transgene did not reduce the expression levels of ADF-specific marker gene *Psrh-142*::*mCherry* or alter ADF morphology (Fig 6C). Further, when GOA-1(S47C) transgenic lines were induced to form dauers or crossed into *tir-1(gf)* background, ADF *tph-1*::*gfp* expression levels were enhanced as compared to non-dauer GOA-1(S47C) transgenic animals (Fig 6B). Like *gpb-1(yz71)* dauers and *tir-1(gf);gpb-1(yz71)* double mutant (Fig 3A and 3D), ADF *tph-1*::*gfp* levels in GOA-1(S47C) transgenic dauers and *tir-1(gf);GOA-1(S47C)* animals were lower than WT dauers and *tir-1(gf)* single mutants (Fig 6B). Combined, these data support our hypothesis that GOA-1(S47C) inactivated GPB-1 in ADF leading to diminished baseline *tph-1*::*gfp* expression. Interestingly, the *Pcat-1*::*goa-1(S47C)* transgene also reduced *tph-1*::*gfp* in NSM. We speculate that NSM *tph-1* expression might also be sensitive to G protein signaling, which was blocked by GOA-1(S47C). These data together endorse the model in which GPB-1-mediated Gβ signaling sets the baseline *tph-1* expression in ADF, and this GPB-1 signaling is controlled by its interaction with GOA-1 (Fig 6D).

### *tph-1* expression in ADF neurons controls specific innate behaviors

Studies of *tph-1* mutants have demonstrated a requirement for 5-HT in a variety of innate and stress-induced behaviors [9,11]. As the first step toward understanding the physiological importance of cell-specific regulation of 5-HT synthesis, we investigated the role of ADF-produced 5-HT in three well-characterized 5-HT-regulated innate behaviors: pharyngeal pumping, locomotion and egg laying. Because of the broad expression and multiple functional roles, disruption of *gpb-1* and *ocr-2* could impair multiple sites in the neural circuits. To identify the role of 5-HT produced in specific neurons, we therefore utilized established transgenic lines expressing *tph-1* either in ADF (*ADF*::*tph-1*) or in NSM (*NSM*::*tph-1*)[11]. We integrated these transgenes onto the chromosomes of *tph-1* deletion mutants and compared them with *tph-1* mutants.

**Fig 7:**
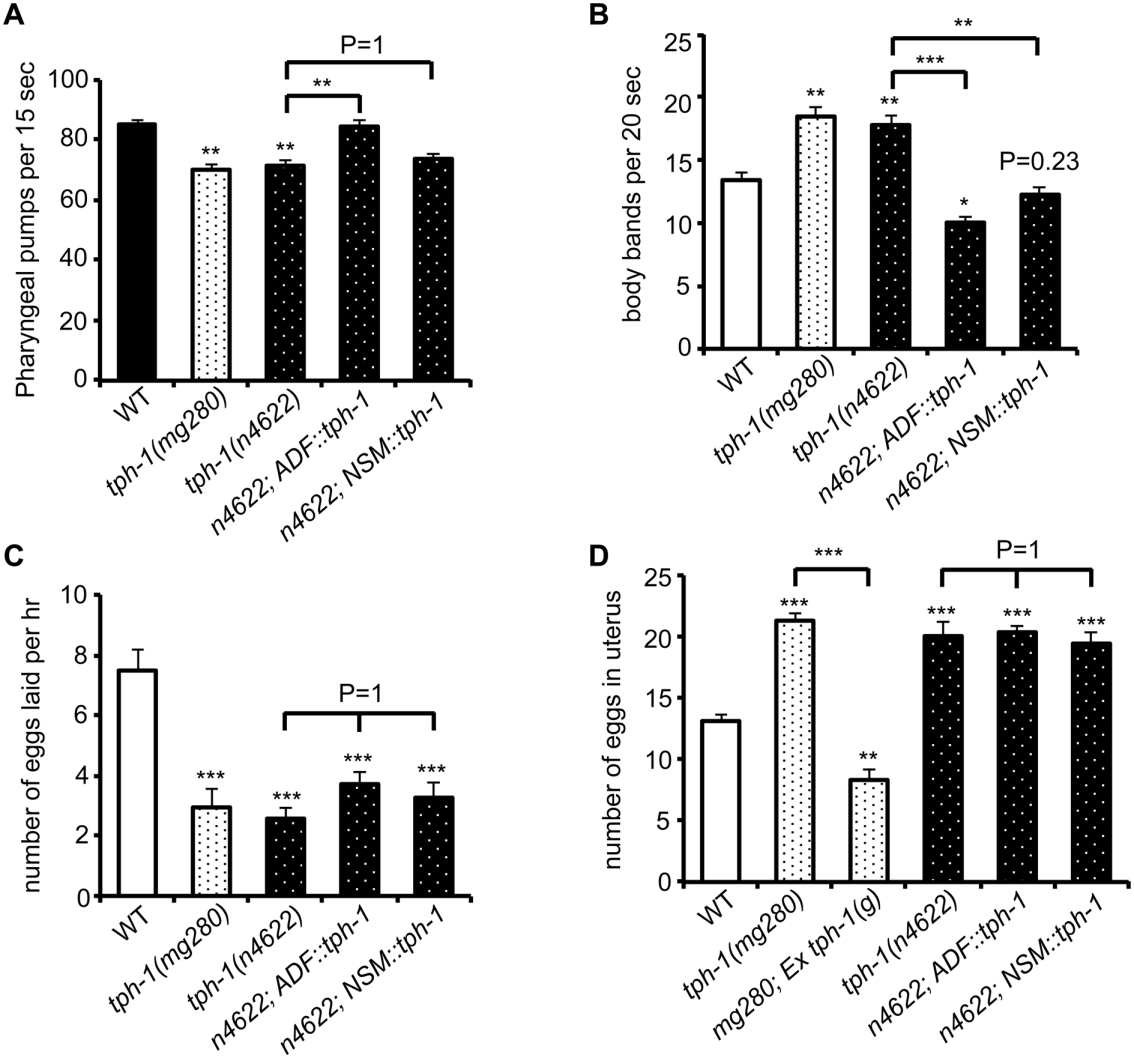
ADF-produced 5-HT modulates specific 5-HT-regulated innate behaviors. **(A)** *tph-1* expressed in ADF not in NSM rescued pumping rates of *tph-1* mutants. **(B)** *tph-1* expressed in ADF and in NSM concerted to reduce locomotory rates. **(C—D**) *tph-1* expressed in ADF and NSM is not required for 5-HT-regulation of egg-laying rates or egg accumulation in the uterus. Data represent the summary of 4–9 trials. Differences between the groups are indicated, and the differences to WT are marked above the bars, ** p < 0.01, *** p < 0.001 (ANOVA followed by Bonferroni test).

*C*. *elegans* feeds continuously on a *E*. *coli* bacterial lawn, by rhythmic contractions of the pharyngeal muscles [33]. The rate of pharyngeal pumping is a measure of *C*. *elegans* feeding behavior and is modulated by food availability and 5-HT [33]. Abundant food or applying exogenous 5-HT stimulates the pumping rates. In the presence of food, *tph-1* null mutants displayed reduced pumping rates compared to WT animals, as previously observed [9]. We observed that the *ADF*::*tph-1* transgene robustly rescued the pumping rate (Fig 7A). In contrast, the *NSM*::*tph-1* transgene did not produce a significant effect (Fig 7A), consistent with the report that laser ablation of NSMs did not reduce the pumping rate [33]. Because ADF senses salts, biotin and nucleotide derivatives [22], ADF-produced 5-HT may modulate the neural circuit that relays attractive signals of nutritious compounds to feeding behavior.

Another *C*. *elegans* rhythmic behavior controlled by both food availability and 5-HT is locomotory rates. *C*. *elegans* slows down on a bacterial lawn, and applying 5-HT inhibits locomotion of WT animals [34,35]. *tph-1* null alleles displayed increased locomotory rates on a bacterial lawn (Fig 7B). Interestingly, both *ADF*::*tph-1* and *NSM*::*tph-1* robustly rescued hyperactive locomotion of *tph-1* mutants (Fig 7B). The pharyngeal NSMs are thought to sense bacteria passing through the pharynx [33], while ADF might sense food signals in the environment. Thus our observations could reflect how 5-HT from distinct neuronal types may converge distinct sensory perceptions to a particular behavioral output.

Because such transgenic arrays are typically overexpressed, we asked whether *ADF*::*tph-1* would alter every 5-HT-regulated behavior, by testing egg-laying behavior. Exogenous 5-HT stimulates the rate of eggs released from the uterus [36]. Conversely, *tph-1* mutants reduce the rate of egg laying and accumulate fertilized eggs in the uterus [9]. Neither *ADF*::*tph-1* nor *NSM*::*tph-1* significantly improved egg-laying behavior, although transgenic expressing genomic *tph-1* sequence fully rescued (Fig 7C and 7D), supporting the notion that the HSN 5-HT-producing motor neurons are connected to the vulva muscles and control egg laying [37]. These results together support a model in which the steady state 5-HT synthesis in ADF tunes the innate behavioral circuits coordinating food intake and food searching movement.

## Discussion

In this study, we identified a cell-autonomous requirement of GPB-1 Gβ signaling for baseline *tph-1* expression in a pair of *C*. *elegans* ADF chemosensory neurons. Genetic analysis suggests that this GPB-1 function is negatively regulated by GOA-1 and acts upstream of the OCR-2 TRPV channel. Behavioral analysis showed that 5-HT produced in specific neuronal types controls defined innate behaviors. We therefore propose that regulation of baseline Tph gene expression under favorite environment represents another layer of transcriptional network that shapes and maintains the serotonergic systems, and could potentially be re-programmed in a cell-specific manner in the mature nervous systems to influence behavior.

### Dedicated transcriptional program defines baseline 5-HT synthesis

Several lines of our data suggest that GPB-1-OCR-2-mediated *tph-1* expression is mechanistically separated from serotonergic fate establishment in ADF neurons. First, *gpb-1(yz71)* mutants did not diminish VMAT CAT-1. Second, the *Pcat-1*::*gpb-1* transgene fully restored the baseline *tph-1*::*gfp* in *yz71* mutants, showing that GPB-1 directs 5-HT synthesis after the serotonergic identity established. Consistently, OCR-2 and its downstream RFX transcription factor DAF-19 are essential for ADF *tph-1* but not *cat-1* expression [12,27]. In mammals, the baseline *Tph2* expression in raphe 5-HT-producing neurons displays a circadian rhythmicity according to glucocorticoid oscillation [38,39]. Conditional knocking out PET-1 in adult mice abrogated *Tph2* but not VMAT expression in specific sets of raphe 5-HT-producing neurons, leading to enhanced anxiety behavior [8]. These findings emphasize cell-specific regulation of *Tph* gene expression in mature neurons as a key to maintain 5-HT signaling in evolutionary diverse organisms.

Our data indicated that cell-autonomous GPB-1 sets the ADF *tph-1* expression baseline. At present, it is unclear how this signaling pathway is activated. This signaling is unlikely to be activated by environmental cues, because cilia mutants that cannot detect external cues do not diminish ADF *tph-1* expression [10]. Accumulating evidence indicates that G proteins can be activated by receptor-independent cell intrinsic cues [16,17]. While further studies are required to define the GPB-1 activation mechanism, GPB-1-regulation of *tph-1* differs from the mechanisms that maintain the terminally differentiated neuronal cell identity [5], as GPB-1 is not essential for preserving ADF identity or axon/dendritic architecture. Thus, the GPB-1-OCR-2 signaling pathway in ADF is likely to represent another layer of transcriptional network that is dedicated to direct baseline neurotransmitter synthesis in specific neuronal types. Although genetic dissection of 5-HT production in mammalian CNS remains not feasible, evidence has been mounting that genetic variants that reduce *Tph2* expression attend quantitative effects on 5-HT-modulated behavior in rodents and humans [2].

### Gβ GPB-1, not Gα, is a key regulator of *tph-1* baseline expression in ADF neurons

Genetic dissection of transcriptional regulation of 5-HT synthesis in this work took advantage of the viable GPB-1(G162E) reduction-of-function allele. Analysis of the baseline and pathogen-induced *tph-1*::*gfp* expression has revealed two mechanistically separable mechanisms of GPB-1. Our cell-specific rescue experiments, combined with the report that G_q_α EGL-30 function in AWC and AWB neurons is necessary and sufficient for PA14-induced ADF *tph-1* upregulation [25], argue that GPB-1 is a regulator for EGL-30 signaling in AWC and AWB that relay infection cues to ADF. In contrast, GPB-1 regulation of the *tph-1* baseline expression depends on G_o_α GOA-1.

Like GPB-1, GOA-1 is broadly expressed and has been shown to regulate mitotic spindle positions in embryogenesis as well as a variety of behaviors [40]. The role of GOA-1 in regulating baseline *tph-1* expression, however, differs from all these examples in which GOA-1 signaling is essential. In contrast, both *goa-1* deletion and hyperactive GOA-1(Q205L) conferred elevated ADF *tph-1*::*gfp*. These data, together with the results that *ocr-2* and GPB-1(G162E) diminished ADF *tph-1*::*gfp* in the *goa-1* deletion and GOA-1(Q205L) mutants, point to GOA-1 as a negative regulator of the GPB-1-OCR-2 signaling pathway that drives baseline *tph-1* expression (Fig 6D). However, we do not eliminate the possibility that GOA-1 signaling can transduce other sensory cues to influence 5-HT synthesis.

How might G162E disrupt Gβ function? We found that, similar to GPB-1(G162E), expressing GOA-1(S47C), analogous to the mammalian G_o_α(S47C) that is locked in the GDP-bound conformation, diminished ADF *tph-1*::*gfp*. Thus one possibility is that GPB-1(G162E) promotes the heterotrimeric complex in the inactive conformation. Modeling GPB-1 sequence over the G_i_α_1_β1γ2 heterotrimer crystallographic structure revealed that G162 is located at the Gβ surface that binds to Gα switch II helix. GTP-binding of Gα triggers an 120° rotation of the switch II helix, thereby switching on the G protein signaling [28,41]. Thus G162E could block switch II helix rotation. Alternatively, G162E might alter the conformation of nearby G202 and G203 in the switch II helix critical for GTP binding [32], reducing the GTP affinity. These models are also consistent with GPB-1(G162E) abrogation of EGL-30-dependent pathogen-induced *tph-1* upregulation, indicating that G162E obstructs a key point of GPB-1/Gα interaction. However, these models do not address how GPB-1(G162E) diminished *tph-1*::*gfp* induced by GOA-1(Q205L), which is locked in the GTP-bound conformation. As the transgenes form multiple copies of gene arrays, expressing GOA-1(Q205L) driven by the *goa-1* promoter could reduce endogenous *goa-1* transcription due to promoter titration. In addition, GOA-1(Q205L) protein could compete with endogenous GOA-1 for the trafficking machinery and functional sites to liberate GPB-1 as well as GPB-1(G162E). Thus, another possibility would be that G162E also impairs effector signaling that regulates *tph-1* expression.

There is increasing evidence that Gβ interacts directly with a wide range of effectors to modulate diverse downstream cellular responses [16,17]. It is interesting to note that the first example was the activation of a cardiac potassium channel by purified Gβ [16]. A common lesson learnt from X-ray crystal structure determination, NMR analysis and biochemical analysis is that Gβ effector-binding sites are masked by Gβ/Gα switch II binding [17]. In this regard, Gβ-Y145 is a key residue in one such effector-binding hotspot and sits closely to G162 in the crystal structure [17] (Fig 5Biii). Possibly, GDP-bound GOA-1(S47C) locks the switch II into the conformation unable for effector binding to GPB-1, whereas GPB-1(G162E) produces a dual disruption on Gα switch II conformational changes and Gβ effector binding that defines the *tph-1* baseline, thus steady state 5-HT synthesis, in the ADF neurons (Fig 6D).

## Methods

### Strains

*C*. *elegans* strains were maintained at 20°C on NGM agar plates seeded with a lawn of *E*. *coli* OP50 as a food source [42]. WT animals were Bristol strain *N2*. The Hawaiian isolate *CB4856* was used in genetic mapping of *gpb-1(yz71)* mutation. Mutant strains used in this study were: *che-2(e1033)*, *eri-1(mg366);lin-15B(n744)*, *gpb-1(ok1875)/mIn1 [mIs14 dpy-10(e128)]*, *goa-1(n363)*, *ocr-2(yz5)*, *tir-1(yz68)*, MT15434 *tph-1(mg280)*, and *tph-1(n4622)*. Transgenic strains were: *CX6741: tph-1(mg280);Ex[ADF::tph-1]*[11], *CX7749: tph-1(mg280);Ex[NSM::tph-1]*[11], *tph-1(mg280); yzEx126[tph-1(g); Rol-6]* [9], *GR1333: yzIs71[tph-1::gfp; Rol- 6(d)]*[9], *Is[cat-1*::*gfp]*[23], *PS1493*: *dpy-20(e1362);syIs9[pJMG*_*o*_ *QL; Dpy-20(+)]* [31], and *yzEx010[Psrh-142::mCherry;elt-2::gfp][43]*.

### Identification of *gpb-1(yz71)* mutation

*yz71* was isolated from a genetic screen for mutants with diminished *tph-1*::*gfp* in the ADF neurons after ethyl methane sulfonate mutagenesis of GR1333 animals as described previously [12]. Genetic mapping using single-nucleotide polymorphisms (SNP) of *CB4856* localized *yz71* to a 700 kb contig on LG II. Sequencing *yz71* genomic DNA revealed two mutations in this contig. After the two mutations separated by backcrossing with N2, diminished ADF *tph-1*::*gfp* segregated only with the G-to-A transition causing a G162E substitution in the third predicted WD40 repeat of the Gβ protein GPB-1. The G-to-A transition abolished a restriction enzyme BspE1 cutting site, which was used as another method to validate the *yz71* mutation.

### Generation of transgenic animals

All constructs were generated by PCR. *gpb-1(g)* was a genomic DNA fragment encompassing 2,027 bp 5’-noncoding sequence, exons, introns and 933 bp 3’-UTR of the *gpb-1* gene, similar to previously reported [19]. To express *gpb-1* in specific cells, *gpb-1* cDNA sequence was inserted between a heterologous promoter and *unc-54* 3’-UTR. *gpb-1* cDNA was amplified from cDNA mixture prepared from total RNA of WT animals. The following published promoter sequences were used: 1.3 kb *rab-3* promoter expressed in all neurons [44], 2.9 kb *ges-1* promoter expressed in the intestine [45], 3.7 kb *daf-6* promoter expressed in hypodermis and glia [46], 2.7 kb *che-2* [47] and 1.8 kb *dyf-1* [10] promoters both expressed in all ciliated neurons, 2.7 kb *odr-3* promoter expressed in ciliated sensory neurons AWA, AWB, AWC, ADF, ASH, PHA and PHB [48], 4.1 kb *lin-11* genomic fragment encompassing 623 bp promoter to 14 bp of exon 5 expressed in ADF and ADL [49], with a stop codon and SL2 sequence inserted between the *lin-11* and *gpb-1* cDNA sequences, 4.6 kb *cat-1* promoter expressed in monoaminergic neurons [27], 3.4 kb *srh-142* promoter expressed in ADF [43], 2.4 kb *odr-1* promoter expressed in AWB and AWC neurons [50], and 2.6 kb *sra-6* promoter expressed in ASH, ASI plus a few unidentified neurons and intestine [51].

To overexpress GOA-1(S47C) in serotonergic neurons, *goa-1* cDNA was amplified from WT cDNA mixture, and the codon Ser47 (TCG) was changed to Cys47 (TGT), using PCR primer ggagaatcaggaaaa**tgt**actattg, and the mutagenized sequence was fused to the *cat-1* promoter sequence and *unc-54* 3’-UTR.

For generating transgenic worm lines, individual constructs from three independent PCR reactions were pooled to reduce potential PCR errors, and the pooled PCR products were purified (Qiagen) and microinjected into WT or *yz71* mutants carrying an integrated *tph-1*::*gfp*. For several transgenic lines, a construct was first injected into WT animals and then crossed into *yz71* mutant background. The plasmids containing either *elt-2*::*gfp* or *unc-122*::*rfp* were co-injected as a transgenic marker. Typically, two transgenic lines from one injection were analyzed, and data from one representative line are presented. For overexpressing GOA-1(S47C), DNA from two independent preparations were individually injected, and transgenic lines from both injections are presented.

### RNAi

RNA-interference (RNAi) experiments were performed in the background of *eri-1;lin-15B* to enhance RNAi efficiency in neurons, as previously described [12]. RNAi assays were carried out by feeding worms an *E*. *coli HT115* clone expressing dsRNA of a target gene or the control empty L4440 vector (Ahringer RNAi library, University of Cambridge, England). RNAi clones were individually cultured overnight in Luria broth containing 100 μg/ml ampicillin. 600 μl of bacterial culture were spread evenly to cover the surface of assay plates containing NGM medium supplemented with 6mM IPTG and 25μg/ml carbenicillin, and incubated overnight at room temperature. Eggs from *eri-1;lin-15B* animals carrying *tph-1*::*gfp* were placed onto each plate, incubated at 20°C, and GFP levels in ADF of resultant L4 and second day adults were quantified.

### Quantification of *tph-1*::*gfp* expression

The expression of a chromosomally integrated *tph-1*::*gfp* reporter in ADF or NSM neurons in living worms under optimal growth conditions, during pathogen PA14 infection, or in the dauer stage was evaluated by measuring GFP fluorescence intensity, as we have done previously [10,12]. Briefly, images of individual neurons were captured under a 40x objective lens at a fixed exposure time, using an AxioImager Z1 microscope equipped with proper filters and AxioCam MR digital camera (Zeiss, Northwood, NY). The external contour of the cell body in the images was delineated, and fluorescence intensity within the entire cell body was measured using the ImageJ software (National Institute of Health, Maryland).

For quantifying *tph-1*::*gfp* intensity during pathogen infection, first day young adult worms were transferred to standard slow killing assay plates seeded with either PA14 culture [52] or control plates seeded with OP50, incubated at 25°C for 6 hr, images of the ADF neurons were captured and GFP intensity quantified.

For quantifying *tph-1*::*gfp* intensity in dauers, WT and mutants were induced to form dauers by dauer pheromones and high growth temperature at 25°C as previously described [10,12]. Gravid adults from each strain were transferred to NGM plates supplied with dauer pheromone, allowed to lay eggs in a 25°C incubator, the adults were then removed from the plates, and dauers developed from hatched eggs on the plates were analyzed 3 days later. For each strain the value of dauers was compared to that of L4 sibling grown on NGM plates without pheromone and assayed on the same day. For some experiments, starvation was used as a second method to induce dauer formation. Data from dauers induced by the two conditions are comparable.

Data represent the average of at least three trials unless specified otherwise. For each trial, 15–25 animals per genotype per condition and treatment were analyzed and compared to the controls assayed on the same day. WT animals under the same conditions and treatments were analyzed for every experiment.

### Indirect immunofluorescence microscopy and dye DiI filling

5-HT immunostaining and quantification of 5-HT immunoreactivity in individual neurons were performed with whole mount worms as we described previously [9], using rabbit antibody against 5-HT (purchased from Dr. H.W.M. Steinbusch, Maastricht University, Masstricht, The Netherlands). The staining patterns were visualized via Alexa Fluor 594 conjugated goat anti-rabbit antibodies (Invitrogen) under an AxioImager Z1 microscope equipped with proper filters. To quantify the intensity of 5-HT immunoreactivity, images of ADF or NSM neurons in individual worms were captured under a 40x objective lens at a fixed exposure time with 100% UV exposure level. For each image, fluorescence intensity of a 10x10 pixels area within a cell body was quantified using the ImageJ software. To exclude staining background, fluorescence intensity over a 10x10 pixels area posterior to the cell body in the same image was quantified, and the value of the background was subtracted from the value of the corresponding neuronal area.

GPB-1 immunostaining was performed according to a published protocol [19] with modifications. Briefly, well-fed mixed stages of worms and embryos were washed off culture plates, rinsed with water, transferred to 0.01% poly-L-lysine-coated slide, crushed by pressing the sample with a coverslip, and frozen on dry ice. The coverslip was then removed and the samples were treated with methanol and acetone. Following serial rehydration, the preparations were incubated with a blotting mix of 0.1% Tween 20 and 5% non-fat milk overnight at 4°C, and stained with rabbit antibody against GPB-1 [20]. The staining patterns were visualized via Alexa Fluor 594 conjugated goat anti-rabbit antibodies.

DiI staining was performed as we previously described [10,12], by soaking well-fed living worms in M9 buffer containing 10 μg/ml DiI for 2 hr, and visualization of the staining pattern under a fluorescence microscope with a proper filter.

### Structural modeling of Gβ(G162E) substitution

WD40 repeats are in reference of published predicted GPB-1 sequence [53]. For homology comparisons, the amino acid sequence of *C*. *elegans* GPB-1, human Gβ1 GNB1 (GenBank: CAG33065.1), human Gβ2 GNB2 (GenBank: CAG46530.1) and bovine β1 GNB1 (NCBI reference sequence: NP_786971.2) were aligned using the ClustalW2 software [54]. The bovine Gβ1 protein shares 100% and 86% amino acid identity to human Gβ1 and *C*. *elegans* GPB-1, respectively.

Crystal structure of the rat G_i_α and bovine β1γ2 heterotrimer (PDB entry: 1GP2)[28] was used to model the G162E substitution with a focus on the interface between Gβ and Gα switch II helix. The public crystal structural data were downloaded from the protein data bank (http://www.rcsb.org/pdb/explore/explore.do?structureId=1GP2, PDB entry: 1GP2), and displayed as cartoon structures using the PyMOL software (http://www.pymol.org). To model Gβ/Gα switch II helix contacting interface, Gβ-G162 and other contacting residues in the Gβ and Gα identified by the crystallography were manually selected, colored and their side chains displayed. To model the G162E substitution, G162 was manually replaced by E in the Gβ sequence, using the Crystallographic Object-Oriented Toolkit (COOT) [55], and modeled again using PyMOL.

### Behavior assays

All behavioral tests were performed with first day young adults, using protocols as we have previously described [9,56], with modifications. Well-fed L4 of WT, mutant and transgenic animals were picked onto fresh plates seeded with a lawn of OP50 as food source, incubated at 20°C, and resultant adults were assayed about 24 hr later. Prior to the assays, animals were allowed to stay at room temperature for 3–5 hr. The rate of pharyngeal pumping was assayed using a stereo dissecting microscope at an 80x magnification. The number of pumps of individual animals within a 15 sec interval was recorded by Moticam 2300 digital camera using the Motic Images Plus 2.0 software (http://www.motic.com/en/index), and the pumping rate in the movie was manually counted. For each assay, 5–12 animals of each genotype were analyzed.

The locomotion rates were evaluated by two methods. The first method measures the locomotory activities by video recording the movement of individual worms on the center of an OP50 lawn on an agar plate in a 30 sec interval, using the Moticam connected to a dissecting microscope. Locomotory rates were determined by counting the number of bends of the anterior body region of individual animals in the movie. Animals that linger around the edge of the bacterial lawn for more than 5 sec were not analyzed. The second method counted the number of the body bends manually under a dissecting scope. 5–15 animals of each genotype were analyzed for every trial.

Egg-laying behavior was evaluated by two assays. First assay measures the number of eggs laid by a worm within a one-hour interval. For each strain, 10 animals were transferred onto a fresh plate, and the number of eggs laid on the plates was scored one hour later. The second assay determines eggs carried in the uterus of animals that were used for the egg-laying count assay, determining that the reduced number of eggs laid was not simply due to reduced number of eggs in the uterus. To score the number of eggs in the uterus, individual worms were placed into a drop of solution containing 3% of commercial bleach and 1N NaOH on an agar pad on a glass slide. The bleach solution dissolved the body of animals, and eggs, which were protected by their eggshells, were scored immediately. For each behavioral test, the assays were repeated at least 4 times, and representative data pooled from 4–9 trials are presented.

### Statistics

Unpaired Student’s t-test was used for comparisons between a mutant and WT, or between two mutants or two treatments. For comparison between multiple groups, one-way ANOVA followed by Bonferroni test were performed. For comparison between multiple groups with different treatments, two-way ANOVA followed by Tukey’s test were performed.
